# Transient expression analysis of allelic variants of a VNTR in the dopamine transporter gene (*DAT1*)

**DOI:** 10.1186/1471-2156-6-3

**Published:** 2005-01-31

**Authors:** Jonathan Mill, Philip Asherson, Ian Craig, Ursula M D'Souza

**Affiliations:** 1 Institute of Psychiatry, MRC Social, Genetic, and Developmental Psychiatry (SGDP) Centre, De Crespigny Park, Denmark Hill, London, SE5 8AF, UK

## Abstract

**Background:**

The 10-repeat allele of a variable number tandem repeat (VNTR) polymorphism in the 3'-untranslated region of the dopamine transporter gene (*DAT1*) has been associated with a range of psychiatric phenotypes, most notably attention-deficit hyperactivity disorder. The mechanism for this association is not yet understood, although several lines of evidence implicate variation in gene expression. In this study we have characterised the genomic structure of the 9- and 10-repeat VNTR alleles, and directly examined the role of the polymorphism in mediating gene expression by measuring comparative *in vitro *cellular expression using a reporter-gene assay system.

**Results:**

Differences in the sequence of the 9- and 10- repeat alleles were confirmed but no polymorphic differences were observed between individuals. There was no difference in expression of reporter gene constructs containing the two alleles.

**Conclusions:**

Our data suggests that this VNTR polymorphism may not have a direct effect on *DAT1 *expression and that the associations observed with psychiatric phenotypes may be mediated via linkage disequilibrium with other functional polymorphisms.

## Background

The dopamine transporter (DAT) mediates uptake of dopamine into presynaptic neurons, and is a major target for various pharmacologically active stimulants such as cocaine. Genetic association studies provide considerable evidence that a variable number tandem repeat (VNTR) polymorphism in the 3'-untranslated region (UTR) of the dopamine transporter gene (*DAT1*) is associated with a range of psychiatric phenotypes. In particular, the 10-repeat allele of this polymorphism has been widely associated with attention deficit hyperactivity disorder (ADHD) (e.g. [1,2]), although there have been several non-replications reported (e.g. [3,4]). The mechanism for this association is not yet understood, although several lines of evidence implicate variation in gene expression [5]. The VNTR polymorphism consists of a 40 bp sequence that most frequently occurs as 9 or 10 tandem repeat units, although 3 through to 11 repeats are also observed. Its location within the transcribed 3'-UTR is interesting since these regions have been shown to play an important role in the regulation of transcription efficiency, mRNA stability or mRNA sub-cellular localization [6].

Several *in vivo *studies using single photon emission computed tomography (SPECT) show an increased density of DAT in ADHD probands compared to controls [7-10], although such findings are not ubiquitous [11]. In addition, several studies suggest there may be an association between VNTR genotype and DAT density [e.g. [12,13]]. Furthermore, methylphenidate, which is used as a treatment for ADHD, has been shown to lower levels of DAT in the brain [9]. Finally, the most thorough investigation of methylphenidate response in relation to *DAT1 *genotype suggests that the 10-repeat allele is associated with a positive response to the drug [14] as would be expected if the density of the DAT, to which methylphenidate binds, is increased in individuals with the 10-repeat allele. Two smaller studies also report an effect of the *DAT1 *VNTR on methylphenidate response, although they find poor response is associated with the 10-repeat allele [15,16]. Previously, we measured *DAT1 *messenger RNA (mRNA) levels in cerebellum, temporal lobe and lymphocytes and observed that increased levels of *DAT1 *expression were associated with the number of 10-repeat alleles [17]. These data suggested that the VNTR or another polymorphism in linkage disequilibrium (LD) with the VNTR is involved in regulating expression of this gene.

A number of groups have investigated the functional role of the *DAT1 *VNTR *in vitro*, although the results from these studies are inconclusive. Michelhaugh et al demonstrated that the human *DAT1 *9-repeat VNTR enhances transcription in SN4741 cells, obtained from a mouse-embryonic substantia nigra-derived cell line [18]. However, their study did not compare the effect of the 10- versus the 9-repeat. Furthermore, their constructs contained the repeat region inserted upstream of the reporter gene promoter, and not in the 3'UTR of the gene itself. Thus the position of the repeat did not mirror that seen in the wild-type *DAT1 *gene. It has been postulated that large repeat motifs in the 3'UTR of genes may alter mRNA stability, but such effects would not be detected in the research presented by Michelhaugh et al. Furthermore, it has been shown that the enhancing effect of sequence elements is largely determined by their relative location [19]. Fuke et al examined the effect of the VNTR polymorphism on gene expression using the luciferase reporter system in COS-7 cells [20]. They found that luciferase expression was significantly higher in cells transfected with vectors containing the 10-repeat allele compared to the 7-repeat or 9-repeat alleles. However, they used a monkey cell-line that may not be representative of endogenous human cells and did not employ an internal transfection control for the numerous forms of experimental variability that could affect their data. Miller and Madras concluded that the 9-repeat allele was correlated with increased expression in HEK-293 cells compared with the 10-repeat, but that expression was further mediated by a SNP also located in the 3'UTR of *DAT1 *[21]. Again, this study did not utilise the dual-luciferase assay system and thus their data could be skewed by experimental variability. Inoue-Murayama et al investigated the functional effect of *DAT1 *VNTR alleles from several primate species transfected into the human neuroblastoma cell-line, SK-N-SH [22]. They found that the VNTR sequences of nonhuman primates show higher reporter-gene activity compared to human alleles, and a general trend for longer VNTR alleles to reduce transcription. Finally, Greenwood & Kelsoe found no effect on transcription of the 9- and 10-repeat alleles in SN4741 cells, but found that introns 9, 12, and 14 may contain enhancer elements capable of increasing expression ~2-fold [23]. They conclude that it may be the particular combination of polymorphisms in a haplotype across the gene that ultimately effects *DAT1 *gene expression.

The aim of this study was to examine directly the role of the VNTR polymorphism in mediating gene expression by measuring comparative *in vitro *cellular expression implementing a reporter-gene assay system in which the VNTR has been inserted in an appropriate 3' configuration, and using human cell-lines known to express *DAT1*. Analysis was limited specifically to the VNTR sequence itself, so that any functional effect detected could be attributed solely to this candidate polymorphism. Another aim of this study was to fully characterise the genomic structure of the *DAT1 *VNTR. It is possible that any functional effects of the VNTR are not mediated by repeat length, but instead by variation in the actual sequence of the repeat units. Recent work on the *DRD4 *exon III VNTR sequence has demonstrated that there can be considerable variation within the sequence of large VNTRs [24]. Such variation, if present, could confound analyses of gene expression and negate associations with disease based solely on repeat length.

## Results

### Sequence analysis of the DAT1 VNTR

**Figure 1:**
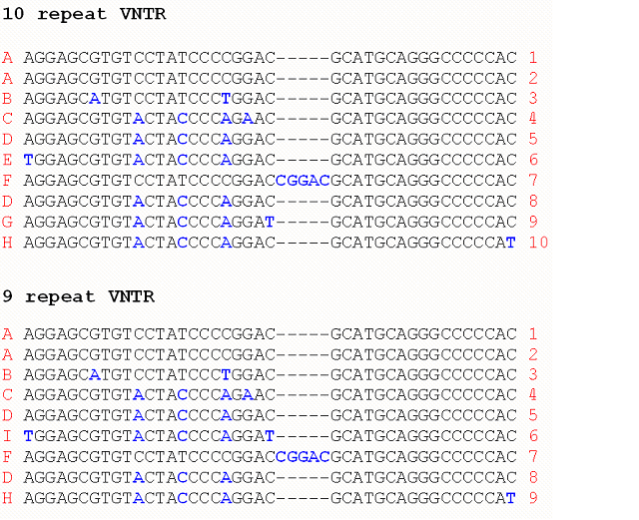
Alignment of the repeat motifs in the 9 and 10 repeat VNTR alleles. Blue letters highlight deviations from the 'A' sequence motif. These sequences are identical to those observed in a Japanese population by Fuke et al [20].

Differences were found between repeat motifs (the individual repeat units making up the total VNTR), and nine variants (labelled A-I) were detected in total (see Figure 1). Interestingly, although differences were found between the orders of motifs in 9- and 10-repeat alleles, they were identical for all examples of each allele size. The order of motifs in the 10-repeat VNTRs was found to be A-A-B-C-D-E-F-D-G-H, and the order in 9-repeat alleles was found to be A-A-B-C-D-I-F-D-H. These sequences are identical to those reported by Fuke et al in a Japanese sample [20]. Furthermore, aligning these sequences up against those available on the NCBI database (accession numbers NM_001044 and AF119117.1) also suggested a lack of between-individual polymorphisms within the VNTR. Interestingly, the 'I' motif (seen in the 9-repeat VNTR but not the 10-repeat) appears to be a combination of the 'E' and 'G' motifs (seen in the 10-repeat VNTR but not in the 9-repeat). The 'F' motif, which occurs in both the 9- and 10-repeat alleles, is also interesting in that it contains 45 base-pairs rather than 40. Furthermore, almost all the between-motif variation occurs within the first (1–24 bp) section of each sequence, before the location of the 5 bp insertion in motif 'F'.

### Luciferase activity from transiently transfected cells

Table 1 shows the luciferase transcriptional activity of the seven transfected pGL3 plasmid constructs in SH-SY5Y and HEK-293 cells. Plasmids containing the VNTR insert produced slightly less transcriptional activity than the original pGL3-promoter and pGL3-control plasmids in both cell-lines, although this difference was not statistically different. No statistical difference in transcriptional activity was detected between constructs containing the 9 or 10-repeat *DAT1 *VNTR sequence in either the SH-SY5Y cells or the HEK-293 cells, although a slight decrease in expression was noticed with increasing repeat length (no insert > 9-repeat insert > 10-repeat insert). Some slight differences were noted between the two cell lines: in both SH-SY5Y cells and HEK-293 cells, the pGL3-basic plasmid, which contains neither promoter nor enhancer sequences, generated the lowest transcriptional activity as expected. In the SH-SY5Y cells, control plasmids containing both an enhancer and promoter showed significantly higher levels of transcriptional activity than the promoter plasmids, although this difference was not seen in the HEK-293 cells.

**Table 1:** Luciferase transcriptional activity of the *DAT1 *VNTR constructs in SH-SY5Y and HEK-293 cells.

**pGL3 Construct**	**Luciferase/*Renilla *ratio (95%CI)**	
	**SH-SY5Y**	**HEK-293**
*pGL3-Basic*	0.0033 (0.0026–0.0041)	0.1811 (0.1197–0.2424)
*pGL3-Control*	1.0407 (0.8464–1.2350)	1.7432 (1.1692–2.3172)
*pGL3-Control-9rpt*	0.7400 (0.6018–0.8781)	1.4963 (1.0416–1.951)
*pGL3-Control-10rpt*	0.5557 (0.4598–0.6515)	1.2098 (0.8637–1.5559)
*pGL3-Promoter*	0.1624 (0.0853–0.2396)	2.3461 (1.246–3.4462)
*pGL3-Promoter-9rpt*	0.1350 (0.1053–0.1646)	1.5967 (1.0963–2.0972)
*pGL3-Promoter-10rpt*	0.1172 (0.0929–0.1416)	1.2916 (0.925–1.6583)

## Discussion

The aims of this study were twofold: first to characterise the genomic structure of the *DAT1 *VNTR; and second to investigate the effect of the 9- and 10-repeat VNTR alleles on levels of transcription. It is possible that any effects, both on behavioural phenotype or levels of *DAT1 *expression, commonly associated with the VNTR are due to polymorphisms *within *the repeat rather than the actual length of the VNTR itself. This theory has often been postulated to explain cases of non-replication of association studies with complex disorders. Recent work on the *DRD4 *exon III VNTR polymorphism has highlighted the existence of both within individual and between individual variation in repeat motif sequence [24]. In other words the 1^st ^and 2^nd ^repeat motifs *within *a certain length *DRD4 *VNTR allele may differ in sequence in a single individual, but there is also variation within the 1^st ^motif between different individuals. We sequenced the *DAT1 *VNTR in individuals homozygous for either the 9- or 10-repeat allele. Differences were found between repeat motifs in both the 9- and 10-repeat alleles, and nine variants were detected in total. Interestingly, although differences were found between the 9- and 10-repeat alleles, the order of the motifs was identical for all examples of each allele size. Given that not one variation in these sequences was observed in the 60 chromosomes sequenced, it can be concluded that any between individual variations that do occur are extremely rare and unlikely to be the cause of common disorders such as ADHD.

Previously, Ueno et al screened the *DAT1 *3'UTR for novel polymorphisms in a Japanese population, but only detected a G>A SNP located upstream of the VNTR at position 2319 [25]. Fuke et al have also sequenced the *DAT1 *VNTR in a small number of Japanese subjects with results identical to those we describe [20]. Miller et al report a SNP that abolishes a *Dra*I restriction site, which they claim to be within the 10-repeat allele [26]. Closer inspection of their paper, however, shows that this polymorphism is located outside of the repeat region and thus their conclusion that they have found a 'novel variant of the 10-repeat allele' is technically incorrect. It is possible, however, that either this polymorphism or that discovered by Ueno et al [25] may be the real risk variant, and associations reported for the VNTR polymorphism may be a result of LD relationships with either of these SNPs.

The lack of between-individual variation in the sequence of the *DAT1 *VNTR is perhaps surprising given the number of polymorphisms seen in other large VNTRs (e.g. [24]). VNTRs are often mutation hotspots with a high level of genetic recombination due to misaligned repeat units that can cause variations in sequence as well as length [27-29]. Furthermore, according to several estimates of SNP frequency across the genome (e.g. [30]), the average total repeat length of ~400 bp might be expected to contain at least one SNP, especially as it is not located in a highly-conserved coding-region of the gene. The fact that there appears to be no between-individual variations within the 9- and 10-repeat VNTR sequences suggests that it may be subjected to some form of selective pressure, and perhaps have an important functional role. A clue to the importance of the *DAT1 *VNTR may come from its location within the 3'UTR of the gene. Sequence motifs in the 3'UTR have been shown to have important roles in translation, mRNA stability, subcellular localization, and polyadenylation [6]. Alternatively, given that the between-motif variations seen within the VNTR are ubiquitous, it is possible that they are relatively ancient and have been pushed towards fixation within the population via demographic and random genetic drift.

The second aim of this study was to examine *in vitro *possible functional consequences of the *DAT1 *VNTR polymorphism. We made constructs containing the 9- and 10-repeat alleles of the DAT1 VNTR and the luciferase reporter gene, and transiently transfected them into SH-SY5Y and HEK-293 cells. Constructs containing the VNTR alleles gave slightly lower levels of luciferase activity compared to vectors without the inserts. Although these differences were not statistically significant, they appear to go against the conclusions of a previous study in which the VNTR sequence acted as a strong enhancer of transcription [18]. However, the trend of our results do agree with data presented by Fuke et al who found that vectors containing the VNTR polymorphism gave lower luciferase expression levels compared to positive control vectors having no VNTR inserted [20]. Furthermore, Greenwood & Kelsoe found no evidence to suggest that the VNTR sequence acts as a transcriptional enhancer [23]. The discrepancies between these results may be explained by the different methodological strategies employed. Michelhaugh et al utilised GFP as a reporter gene [18], which does not have the sensitivity or specificity associated with the luciferase reporter system, and is generally used in qualitative detection assays. Furthermore, they cloned the VNTR sequence upstream of the SV40 promoter, in a location not analogous to the 3'UTR location of the polymorphism in the DAT1 gene. The importance of the location of regulatory elements has been well-documented [19] and so it is possible that the enhancing effect reported by Michelhaugh et al [18] is specific to the location of the VNTR in their particular experimental design. Additionally, it is possible that the VNTR polymorphism has an important role in mediating processes such as mRNA stability – such effects will have been missed by Michelhaugh et al, as their insert is not transcribed.

We found no significant differences in luciferase activity between constructs containing the 9- or 10-repeat *DAT*1 VNTR alleles. Other transient expression studies that have compared the VNTR alleles have provided mixed results. Fuke et al found that luciferase expression was significantly higher in cells transfected with vectors containing the 10-repeat allele compared to the 7-repeat or 9-repeat alleles [20]. Their study used a different cell-line, COS-7, which is derived from African Green Monkey kidney. Miller and Madras, on the other hand, concluded that the 9-repeat allele was correlated with increased expression in HEK-293 cells, but that expression was further mediated by a SNP also located in the 3'UTR of *DAT1 *[21]. Finally, our data is in agreement with that of Greenwood & Kelsoe, who also found no effect on transcription of the 9- and 10-repeat alleles in SN4741 cells, but found that introns 9, 12, and 14 may contain enhancer elements capable of increasing expression ~2-fold [23].

Therefore, our data suggest that the VNTR polymorphism itself may *not *be functional. Unlike the studies of Fuke et al and Miller & Madras [20,21], our inserts contained no flanking sequence and were restricted to specifically the VNTR itself. There is considerable evidence that there is a functional polymorphism in the vicinity of the 3'UTR of the *DAT1 *gene. Genetic association studies with ADHD, SPECT brain imaging studies, and correlations with levels of DAT protein and *DAT1 *mRNA all suggest that a variant associated with *DAT1 *expression is present in this region. Given that in each of these associations, the VNTR has been nominated as the causative polymorphism, it is likely that the real risk variant is in strong LD with it. Ueno et al and Miller et al both report novel SNPs located within the 3'UTR and close to the VNTR [25,26]. It is possible that either of these SNPs, or another polymorphism yet to be characterised, is mediating expression of *DAT1 *and is the real risk variant.

There are several obvious limitations to this study. First, it is not known how well *in vitro *studies of gene expression reflect patterns seen *in vivo*. Future work could employ animal models to characterise more realistically the effect of the VNTR on *DAT1 *expression. Second, while we ensured that we used cell-lines that naturally express *DAT1*, and inserted the VNTR into the correct 3'UTR location of the luciferase reporter gene, our constructs could have been improved by using a homologous DAT promoter. Third, we only cloned a very small portion of the *DAT1 *gene. Even though this was necessary to examine functional effects specific to the VNTR, the fact that the majority of the *DAT1 *gene was absent means that it is likely that several *cis*-acting regulatory elements were not present in the constructs and thus the observed expression may not reflect the actual regulation of the gene. Finally these studies have only analysed *DAT1 *gene expression in the basal state, and complexities such as the induction of expression by factors such as cellular signals would thus be missed. Future work should focus on systematically characterising the remainder of the 3'UTR to discover the functional effects of other polymorphisms in this candidate region.

## Conclusion

In this study we have characterised the genomic structure of the 9- and 10-repeat *DAT1 *VNTR alleles, and directly examined the role of the polymorphism in mediating gene expression by measuring comparative *in vitro *cellular expression using a reporter-gene assay system. No expression differences were observed between the 9- and 10-repeat alleles suggesting that this polymorphism may not have a direct effect on *DAT1 *function.

## Methods

### Sequence analysis of the DAT1 VNTR polymorphism

Before cloning the *DAT1 *VNTR and performing expression analyses it was imperative to characterise fully its structure. If there was variation within the VNTR between individuals who have repeats of the same length, then these variants could confound expression analyses based simply upon length. Thirty individuals of predominantly Caucasian ethnicity, homozygous for either the 9- or 10-repeat allele of the VNTR, were selected for sequencing. The VNTR region was amplified on an MJ PTC-225 thermal cycler (MJ Research, Massachusetts, USA) using the primers 5'- TGT GGT GTA GGG AAC GGC C-3' and 5'- CAT TCG CAA ACA TAA AAA CTG TTG T-3' using a standard PCR protocol with a proofreading polymerase and an annealing temperature of 58°C. PCR products were run on a 2% agarose gel stained with ethidium bromide, and then purified using the QIAquick Gel Extraction Kit (Qiagen, Crawley, UK). Purified fragments were sequenced using an ABI BigDye Terminator (v3.0) Cycle Sequencing kit (PE Applied Biosystems, Foster City, CA, USA) and electrophoresed on an ABI 3100 Genetic Analyzer (PE Applied Biosystems). Sequencing traces were analysed using Sequencher software (Gene Codes Corporation, Ann Arbor, MI, USA) and multiple samples aligned to aid mutation detection.

### Cloning the DAT1 VNTR into pGL3 gene expression vectors

The pGL3 expression vector family (Promega, UK) contains an *Xba *I restriction site (TCTAGA) immediately downstream from the luciferase reporter gene, enabling inserts to be cloned into the 3'UTR. Primers were designed to directly flank the *DAT1 *VNTR and to both of these an *Xba *I restriction site, along with three extra bases of DAT1 sequence, were added. The primer sequences used were: 5'-TGT TCT AGA TTG TGG TGT AGG GAA CGG C-3' and 5'-AGG TCT AGA AGA GTG TTG GTC TGC AGG CT-3'. The aim of this project was to concentrate solely on the functional significance of the VNTR, so flanking regions around the VNTR were kept as small as possible. Using these primers, the VNTR was amplified in individuals homozygous for the 9- and 10-repeat alleles using standard PCR conditions with an annealing temperature of 55°C. The PCR products were isolated from a 2% agarose gel as described above. The purified fragments were cloned into pCRII TA cloning vectors (Invitrogen, UK), which were then transformed into TOP10F *E. Coli *cells (Invitrogen, UK) following the manufacturers protocol. Colonies containing recombinant plasmids were identified using X-gal and IPTG. DNA from colonies containing recombinant plasmids was prepared using a Qiagen Midi-prep kit (Qiagen, UK), and the presence of insertions was verified using *Xba *I restriction enzyme digestion and further checked using direct plasmid sequencing. The *Xba *I digested 9- and 10-repeat VNTR inserts were run on a 2% agarose gel and purified as described above. The fragments were cloned into pGL3-Control and pGL3-Promoter vectors. The pGL3-Control vector contains both SV40 promoter and enhancer sequences whereas the pGL3-Promoter vector contains only an SV40 promoter. Additionally the pGL3-Basic vector which lacks eukaryotic promoter and enhancer elements was tested on its own without any insert, for transcriptional activity and served as a negative control. Five colonies from each of the four cloning reactions (pGL3-Control-9rpt, pGL3-Promoter-9rpt, pGL3-Control-10rpt, and pGL3-Promoter-10rpt) were selected. Following plasmid DNA preparation, the presence and orientation of the inserts was again verified by *Xba *I digestion and fluorescent sequencing. New DNA stocks for each of the 8 plasmids to be used in subsequent transfection experiments (pGL3-Control, pGL3-Promoter, pGL3-Basic, pRLSV40, pGL3-Control-9rpt, pGL3-Promoter-9rpt, pGL3-Control-10rpt, and pGL3-Promoter-10rpt) were prepared, and tested again by *Xba *I digestion.

### Cell culture, transient transfections, and luciferase assays

SH-SY5Y and HEK-293 cells were purchased from ATCC (VA, USA). The SH-SY5Y cell line is a thrice-cloned subline of the neuroblastoma cell line SK-N-SH. The HEK-293 cell line is derived from human embryonic kidney. A problem with many *in vitro *studies of gene expression using reporter genes is that the cell-lines used do not naturally express the gene of interest (i.e. *DAT1*). Before we transfected our constructs into the SH-SY5Y and HEK-293 cell lines they were tested for *DAT1 *expression using quantitative RT-PCR. Both cell lines were found to naturally express *DAT1 *making them suitable for our experiments (data not shown). In addition previous in vitro studies have shown regulation of the *DAT1 *gene in both the HEK-293 and SH-SY5Y cell lines [31,32], suggesting endogenous expression of the gene in these cell types.

Both cell lines were cultured in 6-well tissue culture plates containing Minimun essential medium Eagle (ATCC) supplemented with 10% foetal bovine serum (Invitrogen, UK). Cells were grown at 37°C in a humidified atmosphere containing 5% CO_2_. Transfection of the SH-SY5Y cells was carried out using a calcium phosphate transfection kit (Invitrogen, UK). The two different transfection methods used were found to be optimal for each line respectively. The HEK-293 cell line was transiently transfected using Lipofectamine 2000 (Invitrogen, UK). Transfection efficiencies were normalised by the co-transfection of the *Renilla *vector, pRL-SV40 (Promega, UK). Following transfection, the cells were allowed to grow for 48 hours. The cells were then washed and 500 μl cell lysis buffer (Invitrogen, UK) was added to each well. The firefly luciferase and *Renilla *luciferase assays were carried out using the Dual-Luciferase Assay System (Promega, UK) following the manufacturers protocol. All transfections were done in triplicate, and repeated at least three times. All comparisons between constructs were analysed by one-way analysis of variance (ANOVA), followed by Tukey post-hoc analysis for pairwise comparisons between specific plasmids.

## Authors contributions

JM carried out the molecular genetic studies, participated in the sequence alignment and drafted the manuscript. UD participated in the overall design and co-ordination of the study with supervision of cell culture techniques. PA/IC contributed to the interpretation of findings and general points of the experimental design. All authors read and approved the final manuscript
